# Exploration of synchrotron Mössbauer microscopy with micrometer resolution: forward and a new backscattering modality on natural samples

**DOI:** 10.1107/S0909049512032414

**Published:** 2012-08-08

**Authors:** Lifen Yan, Jiyong Zhao, Thomas S. Toellner, Ralu Divan, Shenglan Xu, Zhonghou Cai, Joseph S. Boesenberg, Jon M. Friedrich, Stephen P. Cramer, Esen E. Alp

**Affiliations:** a Department of Chemistry, University of California, Davis, CA 95616, USA; b X-ray Sciences Division, Argonne National Laboratory, Argonne, IL 60439, USA; c Center for Nanoscale Materials, Argonne National Laboratory, Argonne, IL 60439, USA; d Biosciences Division, Argonne National Laboratory, Argonne, IL 60439, USA; e Department of Earth and Planetary Sciences, American Museum of Natural History, New York, NY 10024, USA; f Department of Geological Sciences, Brown University, Providence, RI 02912, USA; g Department of Chemistry, Fordham University, Bronx, NY 10458, USA

**Keywords:** synchrotron Mössbauer microscopy, nuclear resonant incoherent X-ray imaging, meteorite, ^57^Fe phantom, 5 µm resolution

## Abstract

New aspects of synchrotron Mössbauer microscopy are presented. A 5 µm spatial resolution is achieved, and sub-micrometer resolution is envisioned. Two distinct and unique methods, synchrotron Mössbauer imaging and nuclear resonant incoherent X-ray imaging, are used to resolve spatial distribution of species that are chemically and magnetically distinct from one another. Proof-of-principle experiments were performed on enriched ^57^Fe phantoms, and on samples with natural isotopic abundance, such as meteorites.

## Abstract 2

New aspects of synchrotron Mössbauer microscopy have been reported, including micrometer spatial resolution, forward as well as backscattering geometry, and the ability to measure samples with natural isotopic abundance, such as meteorites.

## 1. Introduction

Synchrotron-based nuclear resonant scattering techniques, such as synchrotron Mössbauer spectroscopy (SMS) (Sturhahn, 2004 ▶; Smirnov, 1999 ▶; Alp *et al.*, 1995 ▶; Lübbers *et al.*, 1999 ▶; Hastings *et al.*, 1991 ▶) and nuclear resonant vibrational spectroscopy (NRVS) (Sturhahn, 2004 ▶; Chumakov & Rüffer, 1998 ▶; Alp *et al.*, 2001 ▶; Smith *et al.*, 2005 ▶; Seto *et al.*, 1995 ▶; Sturhahn *et al.*, 1995 ▶), involve nuclear resonant transitions of Mössbauer nuclei excited by synchrotron radiation. These techniques are sensitive to the chemical environment of Mössbauer isotopes, and have been effective tools for studying the magnetic and chemical structure of materials, inorganic and organic, crystalline and amorphous alike.

Conventional Mössbauer microscopy (Yoshida *et al.*, 2009 ▶) and synchrotron Mössbauer microscopy (Mitsui *et al.*, 2004 ▶) have been introduced previously. Here we report new aspects of synchrotron Mössbauer microscopy including micrometer spatial resolution, forward as well as backscattering geometry, and the ability to measure samples with natural isotopic abundance, such as meteorites. There are two distinct and unique signals that are available to resolve the spatial distribution of Mössbauer isotope-containing species. These are:

(i) Synchrotron Mössbauer spectroscopy imaging (SMS imaging), which collects coherent nuclear resonant scattering signal in the forward direction, and yields information based on hyperfine interactions of the nuclei in the solid;

(ii) Nuclear resonant incoherent X-ray imaging (NRIX imaging), which collects incoherent nuclear resonant scattering in the 4π direction, could provide lattice dynamics information, and allows study of thick samples like natural rocks.

In both SMS imaging and NRIX imaging the time-integrated signal can be recorded point by point to reveal the distribution of Mössbauer isotope species. Then getting closer to the interesting spots, with SMS imaging, the time-dependent spectrum can reveal hyperfine interactions, leading to information about the magnetic, valence and spin state of the system, and local atomic symmetry. With NRIX imaging, by tuning the incident X-ray energy off nuclear resonance in the 100 meV range, the energy spectrum of phonon excitation probability density could reveal phonon density states, average force constant and kinetic energy. This latter information can be converted to identify isotope fractionation ratios (Polyakov, 2009 ▶).

We performed the proof-of-principle experiments by employing the instruments at beamline 3-ID at the Advanced Photon Source (APS), Argonne National Laboratory (ANL). Excellent signal-to-noise ratio is achieved. Resolution to 5 µm has been achieved, and sub-micrometer is envisioned. Synchrotron-radiation-based nuclear resonant scattering spectroscopy offers greater applicability by further reducing the beam size to commensurate with the needs of diverse experiments of interest in biology, mineralogy, paleomagnetism, geochemistry, and the synthesis of new materials under high pressure.

## 2. Technique related

### 2.1. Experimental set-up

**Figure 1:**
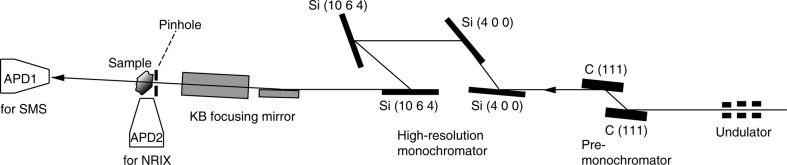
Schematic set-up for SMS imaging and NRIX imaging microscopy, consisting of a pre-monochromator, high-energy-resolution tunable monochromator, focusing optics, pinhole to clean up the beam size and shape, sample translation stages and nanosecond time-resolved detectors for transmission and scattering detection modes.

Fig. 1 ▶ shows the set-up for SMS imaging and NRIX imaging at beamline 3-ID at APS. The sample was placed on a movable stage. The broadband X-ray beam, produced from a 4.8 m-long 2.7 cm-period undulator, is monochromated through two stages. The primary high-heat-load monochromator (HHLM) consists of a pair of high-quality artificial diamond (1 1 1) single crystals with (+ −) arrangement, which reduces the X-ray bandwidth to the eV order around the nuclear resonant energy. The second stage uses a high-resolution monochromator (HRM), by which the X-ray beam is monochromated further to meV bandpass. Each HRM is specifically designed for a specific isotope. For the ^57^Fe isotope, an X-ray beam of 14.4 keV is produced from the undulator in the first harmonic; after HHLM the X-ray bandwidth is reduced to 1.1 eV; the HRM used for the ^57^Fe isotope consists of two Si (4 0 0) followed by two Si (10 6 4) crystal reflections with an ‘in-line’ (+ − − +) scattering geometry, which gives 1 meV resolution at 14.4125 keV (Toellner, 2000 ▶).

Kirkpatrick–Baez (KB) mirrors are used to focus the X-ray beam (Eng *et al.*, 1998 ▶), and an additional pinhole is used to reduce the beam size further (Fischetti *et al.*, 2009 ▶). The KB arrangement mirrors are two elliptically bent mirrors, which are orthogonal to each other, and can individually focus the beam in the vertical and horizontal directions. The size of the X-ray beam focused onto the sample stage without the pinhole was 20 µm × 18 µm (FWHM in horizontal and vertical directions separately). A pinhole with 5 µm diameter was placed right before the sample (∼2 mm) to reduce the spot size. The parameters for the synchrotron source and components are shown in the supplementary information.^1^ In our experiment we use the ^57^Fe isotope, so all the parameters in this paper are selected for this purpose. The flux before the pinhole is 2 × 10^9^ photons s^−1^ in 1 meV bandwidth, and immediately after the pinhole it is 6 × 10^7^ photons s^−1^. The storage ring operation mode at APS is 24 single bunches, with a time spacing of 153 ns. The mean lifetime of the first nuclear excited state of ^57^Fe is 141 ns. So this mode is adequate for ^57^Fe-containing materials or samples containing isotopes with suitable decay times such as ^83^Kr, ^119^Sn, ^151^Eu and ^161^Dy.

As shown in Fig. 1 ▶, two Si-based avalanche photodiode detectors (APDs) are used to collect the nuclear resonant scattering signal. One is placed in the forward direction to measure in transmission geometry, and the other is placed on the side to collect part of the 4π incoherent but resonant scattering. The nuclear resonant scattering signal is discriminated from other forms of scattering in the time domain, as electronic scattering processes are on the timescale of a picosecond or faster (‘prompt’). The pulse duration at the APS is less than 100 ps, while the nuclear resonant scattering signal is on the timescale of 100 ns (‘delayed’). APDs are employed due to their fast time resolution (ns) and large dynamic range of 10^8^ (Baron & Ruby, 1994 ▶; Toellner *et al.*, 1994 ▶; Baron, 2000 ▶; Kikuta *et al.*, 1991 ▶). The typical time resolution of an APD using a 10 mm × 10 mm area silicon diode is about 1 ns (Baron, 2000 ▶). Based on the current set-up and taking into account the shape of the APD output signal, time distribution of the APD output pulses and bunch purity in the storage ring, the starting time for detecting the delayed signal is usually set to around 20 ns. The APD efficiency could be enhanced by rotating it to increase the effective thickness of Si.

In the forward direction the APD collects the coherent nuclear resonant scattering signal. The hyperfine parameters could be extracted from the decayed time spectrum [for example, using the *CONUSS* program (Sturhahn, 2000 ▶)]. With another APD placed on the side and very close to the sample, we collect the delayed incoherent nuclear resonant-induced iron *K*-edge fluorescence signal of 6.4 keV in a large solid angle. In the latter mode, if we stay on the resonant energy, the detected signal can be used to determine the spatial distribution of iron-containing grains. If we tune the incident beam in the range ±100 meV around the nuclear resonant energy with a HRM, we could record an energy spectrum of the phonon excitation probability. From these data the phonon density of states can be extracted [for example, by employing the *PHOENIX* program (Sturhahn, 2000 ▶)]. Both the time spectrum and the phonon density of states provide very unique spectroscopic characters to this X-ray-based microscope.

### 2.2. Phantom preparation

As a proof-of-principle experiment, we have prepared phantom samples to check the validity and sensitivity of this new imaging modality. The ^57^Fe phantom preparation was carried out at the Center for Nanoscale Materials (CNM) at ANL, as follows:

(i) 500 µm-thick silicon wafers were used [p-type, single-side polished, (100), 1–10 Ω cm], which were cut into 1.5 cm × 1.5 cm pieces. The samples were cleaned with a 4:1 (*v*/*v*) mixture of sulfuric acid (95%, ACS reagent grade, Acros Organics) and hydrogen peroxide (30%, ACS reagent grade, Ricca Chemical) for 10 min. The samples were carefully rinsed with 17.5 MΩ cm-resistivity de-ionized water (DIW) and dried with nitrogen and annealed on a hot plate at 473 K for 5 min.

(ii) A thin layer of metal such as Cr (5 nm) and Au (30 nm) was deposited as plate base on the polished side with a Lesker PVD-250 electron-beam evaporator with a Sigma deposition controller. The films were evaporated at room temperature, with a base pressure of 2 × 10^−8^ torr at a deposition rate of 2 Å s^−1^.

(iii) Positive resist S1813 (Microchem) of 1.5 µm was spin coated at 3000 r.p.m. and baked on a hot plate at 288 K for 1 min.

(iv) Exposure with a laser writer LW405 Microtech (GaN laser of 400 nm wavelength) and development for 30 s in 351 developer (Microchem), diluted 1 to 3 in DIW. The resist structure is used as a mold for plating.

(v) Electroplating in 50 m*M* ^57^FeCl_2_ solution.

(vi) To prepare the FeCl_2_ solution, 120 mg ^57^Fe powder was dissolved in 0.4 ml HCl (37%), and then DIW was added (42 ml). By heating on a hot plate at 373 K for several hours, its color turned from colorless to light orange, and then to yellow. As pure FeCl_2_ solution should be light green to colorless, we think the solution might be a mixture of FeCl_2_ and FeCl_3_. The deposition was performed at 323 K with a current density of 1.8 mA cm^−2^ for a deposition rate of ∼0.6 µm min^−1^.

(vii) The resist was removed with acetone.

We produced several ^57^Fe-coated phantoms for imaging study. One pattern had an area of 150 µm × 150 µm, with characters 15 µm wide. Another one was 450 µm × 450 µm with characters of width 45 µm.

### 2.3. Theoretical calculation

For coherent nuclear resonant scattering in the forward direction, without hyperfine splitting of the nuclear resonance, the intensity could be calculated theoretically using the equation given below (Smirnov, 1999 ▶),
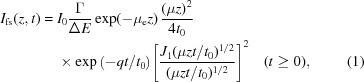
where *I*_0_ is the incident intensity of the synchrotron radiation, Δ*E* is the energy bandwidth determined by HRM, Γ is the natural width of the nuclear excited state, while the characteristic decay time *t*_0_ = 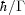. *z* is the X-ray penetration length in the sample, and *J*_1_ is the first-order Bessel function. μ_e_ is the photo-electronic absorption coefficient in the target. μ = σ_0_ *f*β*n* is the linear absorption coefficient at resonance, where σ_0_ is the nuclear resonant absorption cross section, *f* is the Lamb–Mössbauer factor representing the probability for recoilless absorption, β is the isotopic enrichment, and *n* is the number density of nuclear resonant isotopes. *q* describes a broadening of the resonance (Smirnov, 1996 ▶).

Depending on the hyperfine interaction, the degenerated nuclear level will be split into sublevels. There will be multiple nuclear resonances, and the interference between them yields quantum beats. For simplicity, with only two nuclear resonance components ω_1_ and ω_2_, the forward-scattering intensity from a thin layer will be (Smirnov, 1999 ▶)
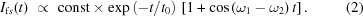



For incoherent nuclear scattering in the 4π direction, the detected intensity could be modeled as (Hu, 1999 ▶) 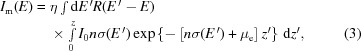
where *R*(*E*) is the energy resolution function of the HRM and σ(*E*) is the energy-dependent nuclear absorption cross section. η is the total detection efficiency, which includes the detector efficiency, solid angle, internal conversion of nucleus decay, fluorescence yield of the *K* shell, atomic scattering factor, *etc.*

To estimate, let *R*(*E*′ − *E*) = 1/Δ*E* only for a small area as |*E*′ − *E*| < Δ*E*/2, otherwise it vanishes. With simple integration of thickness *z*, we can simplify the equation as
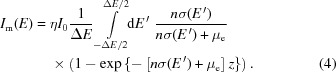
For the elastic peak in the incoherent nuclear resonant scattering, its cross section is given by
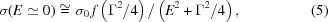
which is much larger than that of electronic scattering.

For the inelastic part of the incoherent nuclear resonant scattering,
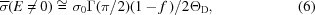
where Θ_D_ is the Debye temperature. The off-resonant nuclear absorption cross section is much smaller than the electronic scattering

Usually the elastic peak is much stronger than the inelastic signal for materials with a recoil-free fraction higher than 10%.

## 3. Measurement results

### 3.1. Phantom-based proof-of-principle test

#### 3.1.1. SMS imaging and time spectrum

**Figure 2:**
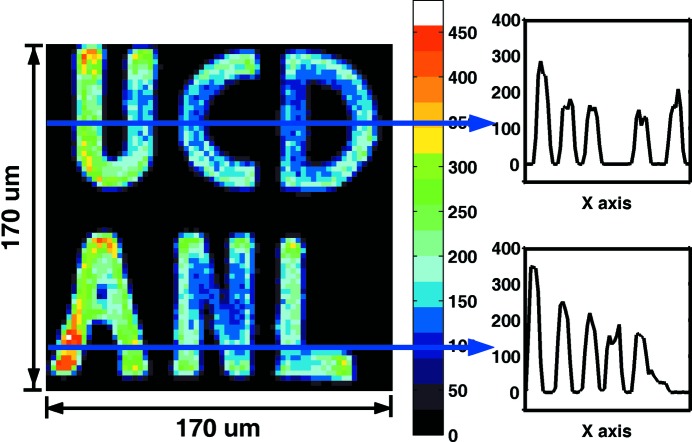
Left: SMS image of the #1 phantom recorded in transmission geometry. Right: intensity distribution along the indicated lines to show the signal-to-noise ratio achieved.

**Figure 3:**
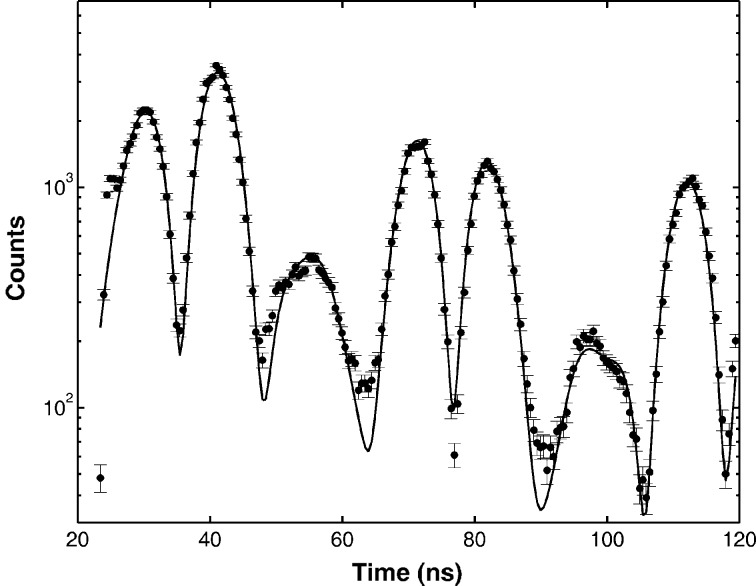
Nuclear decay time spectrum fitted using *CONUSS* software. The solid circles are experimental data, and the solid line is the fitting result from *CONUSS*. *H* = 33.14 T.

The first ^57^Fe phantom we tested had an average thickness of 0.7 µm, with a 150 µm × 150 µm pattern, and width of characters 15 µm. The phantom faced downstream, and one APD was placed in the forward direction. The SMS image was collected (Fig. 2 ▶) and the time spectrum at selected locations was also collected (Fig. 3 ▶). In this measurement the KB mirror and pinhole were both used to reduce the spatial resolution to 5 µm.

As shown in Fig. 2 ▶, the characters in this phantom are displayed clearly with good contrast. Two cross-sectional views are shown on the right-hand side, which display excellent signal-to-noise ratio and indicate the feasibility of studying samples with dilute concentration. The data collection time was 1 s per point, with a step size of 2.5 µm in both directions. The total image was collected in 2 h. During this process the incident energy was kept on resonance within ±0.2 meV.

Fig. 3 ▶ shows the nuclear decay time spectrum collected at a spot in the character ‘N’. In a time window between 23 and 120 ns the detected delayed signal at this spot was 120 counts s^−1^. The delayed time spectrum was fitted using *CONUSS* (Sturhahn, 2000 ▶). The expected thickness at this spot on the letter ‘N’ was 1.0 µm, and the internal magnetic field was simulated to be 33.14 T. Also with *CONUSS* the time-integrated delayed transmission in this time window was calculated as 7.71Γ_0_, where Γ_0_ = 4.67 neV is the natural width of the ^57^Fe nuclear excited state. The electronic absorption is included. With the incident flux as 6 × 10^7^ photons s^−1^ in 1 meV width, considering other factors such as detector efficiency, electronic absorption, *etc.* (listed in Table 1 ▶), we could expect the time-integrated delayed counting rate to be 360 photons s^−1^, which is close to the detected signal. The APD absorbed prompt photon dose is calculated to be 9.7 × 10^6^ photons s^−1^, while the total measured APD counting rate was 5.18 MHz. This discrepancy can be best understood by realising that at high counting rates the APD performs non-linearly (Toellner *et al.*, 1994 ▶).

To guide future experiments, we estimate the theoretical counting rates for two kinds of samples under current conditions with 5 µm resolution. Suppose the APD is set at 85° from normal incidence to optimize its efficiency. With a 0.1 µm ^57^Fe layer on top of a 250 µm Si wafer, we could expect a delayed signal of 20 photons s^−1^. Under these conditions the prompt photon dose absorbed by the APD will be around 2.4 × 10^7^ photons s^−1^. For a 10 µm layer of natural Fe on top of a 250 µm Si wafer, we could expect 50 photons s^−1^ as delayed signal, and an absorbed prompt photon dose of approximately 1.4 × 10^7^ photons s^−1^.

#### 3.1.2. NRIX *versus* SMS imaging

**Figure 4:**
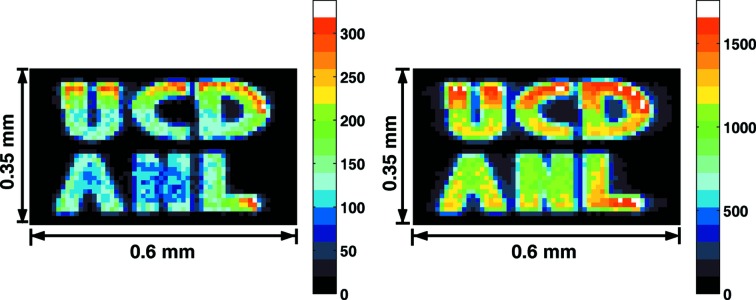
Image of the #2 phantom with NRIX imaging (right), compared with SMS imaging (left).

The second ^57^Fe phantom we chose had an average thickness of 0.6 µm with a pattern area of 450 µm × 450 µm and character width 45 µm. NRIX imaging and SMS imaging were recorded at the same time (Fig. 4 ▶). Only KB mirrors were used to focus the beam without a subsequent pinhole. The phantom faced upstream and had an angle of ∼55° between the X-ray and surface normal. One dimension was shortened because of the rotation. The total data were collected in 40 min, again by scanning 1 s per point, and the step size was 10 µm in both directions.

Fig. 4 ▶ shows that, at the same spot, NRIX imaging has a five times larger signal than SMS imaging. This proves the feasibility of imaging thick samples such as rocks and minerals using the NRIX set-up. This is a salient feature, and makes sample preparation easier, especially for mineralogical or biological samples. We did not record the phonon excitation probability density spectrum with this phantom as it is just pure Fe. However, there is no doubt about this capability, as NRIX at beamline 3-ID is quite a mature technique.

Estimations for both the forward scattering and incoherent scatting were made. The parameters for the forward scattering of the second phantom are listed in Table 1 ▶. The calculated delayed signal was 1150 photons s^−1^, with a photon dose absorbed by the APD of 2.7 × 10^7^ photons s^−1^. At such a high dose APD counting will be saturated (see §4.1). Experimentally we introduced an aluminium absorber to reduce it by an approximate factor of 3. The delayed signal was also reduced by the same factor. The detected APD counting rate was 5 × 10^6^ counts s^−1^, with the maximum detected delayed signal 300 counts s^−1^.

In Table 2 ▶ the parameters that influence the detection efficiency for incoherent scattering were estimated. By employing the HRM resolution function as 1 meV, the numerical integration of equation (4) tends to *I*(*E* ≃ 0) = η*I*_0_ × 3.41 × 10^−5^, with sample thickness 1.1 µm. Taking *I*_0_ = 1.5 × 10^9^ photons s^−1^ meV^−1^ and taking all the efficiency estimations in Table 2 ▶, we found the delayed signal in the incoherent scattering direction to be ∼1600 photons s^−1^, which matches the detected signal well.

#### 3.1.3. Comparison with X-ray fluorescence microscope

**Figure 5:**
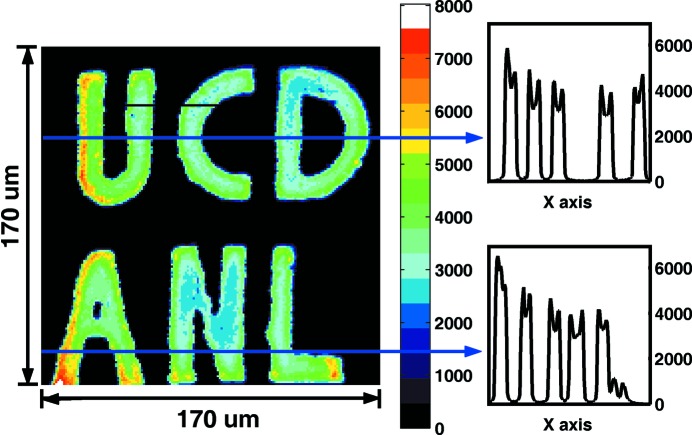
Imaging on the #1 phantom using the X-ray fluorescence technique.

In order to obtain an idea about the effectiveness of our newly introduced imaging methods, we performed X-ray fluorescence imaging on the #1 phantom at beamline 2-ID-D at APS. The beam was focused by a zone plate to 0.2 µm at the sample position. A pinhole with 20 µm diameter was placed before the sample to cut off the beam tails. The flux before the sample was 1.27 × 10^9^ photons s^−1^, and the incident energy was 10.1 keV, with a bandwidth of 2 eV. The collecting channel was set to the *K*_α_ fluorescence of Fe, which is 6.4 keV. The step size was 1.0 µm, the scan area was 170 µm × 170 µm, and the collection time was 0.5 s per point. The image is shown in Fig. 5 ▶. The averaged background is 80 counts in 0.5 s. (The black line in the ‘U’ and ‘C’ was caused by temporary beam loss.)

It is clear that X-ray fluorescence imaging achieves a higher counting rate with higher resolution. This is because it makes use of the full energy bandwidth of the incident beam which allows for much larger flux within a smaller focal spot. Even with this, there are still applications that can benefit from synchrotron Mössbauer imaging despite the low counting rates. As mentioned earlier, SMS imaging and NRIX imaging utilize the properties of nuclear resonant X-ray scattering, and have advantages in distinguishing magnetic properties, valence states, chemical structures and also in the determination of the phonon density of states. With the development of instrumentation and synchrotron sources, there is still potential to increase the delayed signals over the results obtained here.

### 3.2. Meteorite sample test

**Figure 6:**
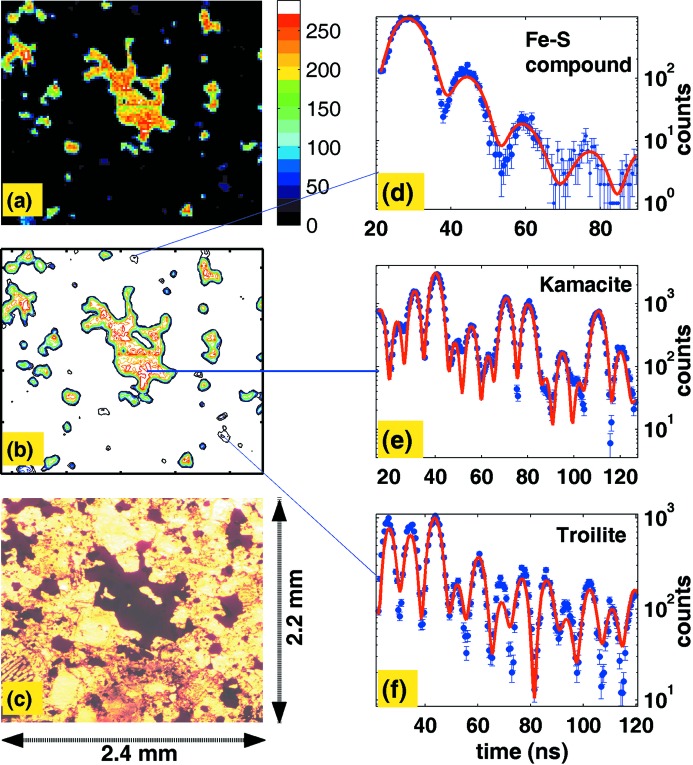
(*a*) Scanning of a petrographic section of the Estacado chondritic meteorite obtained by SMS imaging; (*b*) a contour plot of the same section; (*c*) an optical microscope image of the same area; (*d*–*f*) time spectra for three selected spots, which show (*d*) an iron–sulfur compound grain, (*e*) Fe–Ni alloy and (*f*) troilite FeS.

Some meteorite samples were tested with our nuclear resonant microscope. We show here an image of a petrographic thin section of the Estacado H6 chondrite (Fig. 6 ▶) as a proof-of-principle test. The Estacado sample was obtained from the American Museum of Natural History, and bears the name of its find location: Llano Estacado, Hale County, Texas, USA. A standard petrographic section of thickness 30 µm with 1 mm glass backing was imaged. The left-hand panel of Fig. 6 ▶ shows a comparison of (*a*) the SMS image and (*b*) its contour plot, with (*c*) its optical image. Three typical time domain spectra are displayed in the right-hand panel, which correspond to three grains as indicated. By fitting with *CONUSS*, we found the parameters for these three grains as (*d*) *H* = 16.3 T, QS = 1.29 mm s^−1^; (*e*) *H* = 33.7 T, QS = 0.133 mm s^−1^; (*f*) H = 31 T, QS = 0.35 mm s^−1^. They were assigned to an iron–sulfur compound, kamacite (Fe, Ni alloy) and troilite (FeS) separately.

## 4. Current limits and new methods

### 4.1. APD limit

Our APD set-up only produces one count from each electronic bunch. However, usually there is more than one photon absorbed from each bunch. When μ, the mean number of photons absorbed per bunch by the APD, exceeds 1, the ability to discriminate delayed photons begins to diminish. For the APS 24-bunch mode, μ > 2 occurs when the prompt photon absorption dose in the APD exceeds approximately ∼1.3 × 10^7^ photons s^−1^ (Toellner *et al.*, 1994 ▶). Under current conditions we usually set the starting time of the ‘delayed’ time window around 20 ns, so that this absorption dose does not adversely affect the time-discrimination capability. For such a photon absorption dose, the corresponding APD prompt counting rate is ∼5.6 × 10^6^ counts s^−1^. Even higher doses result in more deterioration of time-discrimination capability, and then it is necessary to reduce this either through the use of an absorber or a pinhole. The necessity to limit the prompt counting rate by using an absorber or pinhole artificially reduces the delayed counting rate as well. This is an inevitable issue for forward-scattering signals. The APD signal from the 4π scattering will not suffer from this problem, as its prompt absorption dose is much less.

### 4.2. Envision of 1 µm resolution

With the 5 µm pinhole, the photon flux incident on the sample was reduced by a factor of 25. This resulted in no loss of delayed signal owing to the fact that it allowed removal of an absorber at the same time. By employing a 1 µm pinhole, the incident flux will be reduced by an additional factor of 25, but the delayed signal will also be affected by this factor. Using better focusing optics instead of a 1 µm pinhole would allow a better resolution without significant loss of delayed signal.

### 4.3. New methods under development

There are some ways to circumvent or decrease the huge prompt photon absorption dose, while not affecting the delayed signal rates. One involves high-speed shuttering (Toellner *et al.*, 2011*b* ▶) which would substantially reduce problems associated with excessive photon absorption rates in the APD. If a high-speed shutter is placed directly before an APD to block the prompt signal, the time-discrimination capability of the APD will not be diminished, thus leading to substantially higher delayed signal rates. One to two orders of magnitude might be expected. Furthermore, using a high-speed shutter would allow other detectors with higher efficiency to substitute the APD. However, as a high-speed shutter requires a very small X-ray beam between sample and detector, it is not practical in the NRIX imaging mode.

Another method involves the development of an ultrahigh-resolution X-ray monochromator. The nuclear level width is of the order of neV. Only photons in this narrow energy range interact with Mössbauer isotope nuclei. Other photons just pass through the sample, resulting in a rather high prompt absorption dose on the APD, which limits the starting time window of the APD. Our current HRM for ^57^Fe has 1 meV bandwidth, filters out broadband photons and has an efficiency of 25% at 14.4125 keV, the ^57^Fe nuclear transition energy. The under-developing high-efficiency ultrahigh-resolution monochromator is expected to have sub-meV bandwidth, and could throw away even more broadband photons (Toellner *et al.*, 2011*a* ▶). With a sub-meV HRM, for forward scattering, the APD could have a broader time window and collect more signal; for incoherent nuclear resonant scattering, higher energy tunability could be achieved.

A multi-element APD (Baron *et al.*, 2006 ▶) is also a good option. With a multi-element APD placed in grazing incidence, the prompt will be distributed amongst the individual elements. Good time resolution and high efficiency can be achieved in the forward direction with this method. For the incoherent nuclear resonant 4π scattering, more signal could be collected owing to a larger solid angle accepted by a multi-element APD.

## 5. Future use

By using these phantoms and a chondritic meteorite sample, we have demonstrated the basic imaging capability of our nuclear resonant microscope, and assessed the practical aspects of such an instrument. We have explored the viability of imaging Fe species. Besides ^57^Fe, this technique can be equally applied to half a dozen other isotopes such as Sn, Dy, Eu, Sm, Sb and Kr. We had success in applying this method to other mineralogical samples, and further results will be presented in another paper.

With these imaging techniques it is possible to image biological samples without radiation damage. For the SMS and NRVS measurements there is no obvious radiation damage that has been detected on biological samples at cryogenic temperature. In these techniques the focused beam flux reaches as high as 10^13^ photons s^−1^ mm^−2^ in 1 meV bandwidth. According to previous studies of radiation damage in biological samples at cryogenic temperatures (Nave, 1995 ▶; Teng & Moffat, 2000 ▶; Sliz *et al.*, 2003 ▶), there are two principal processes of concern: energy deposition caused by photoelectric absorption, and radical reactions induced by thermal diffusion. In our imaging techniques, 14.4 keV X-rays produce modest photoelectric absorption in biological samples, and, with the flux density less than 10^15^ photons s^−1^ mm^−2^, there is both negligible sample heating and insignificant thermal diffusion.

There is potential to increase the nuclear resonant signal while decreasing the total X-ray flux on the sample, by improving the monochromator, or with other improvements such as a high speed shutter. Because only X-rays within the nuclear resonant energy width contribute to the signal, such improvements in the experimental set-up will enhance the measured signal while decreasing, or leaving unchanged, the risk of radiation damage.

## Supplementary Material

Supplementary material file. DOI: 10.1107/S0909049512032414/hf5209sup1.pdf

## Figures and Tables

**Table 1:** Factors affecting the forward-scattering signal for phantoms #1 and #2

Phantom number	Incident flux (photons s^−1^ meV^−1^)	Transmission of Si base on sample	^57^Fe layer transmission due to photoelectric absorption	Time-integrated delayed transmission after ^57^Fe layer	APD efficiency (absorption of Si piece in APD)	X-ray transmission in air (1.0 m from sample to APD)
#1 (APD rotated 80°)	6 × 10^7^ (with pinhole)	0.28 (500 µm)	0.95 (1 µm)	7.62Γ_0_ (1 µm)	0.74 (580 µm)	0.82
#2 (sample rotated 55°)	1.5 × 10^9^ (without pinhole)	0.11 (872 µm)	0.95 (1.1 µm)	8.59Γ_0_ (1.1 µm)	0.21 (100 µm)	0.82

**Table 2:** Detected efficiency estimation for the #2 phantom in the incoherent scattering direction

Internal conversion of nucleus decay	Fluorescence yield for *K*-shell of Fe	Probability of 6.4 keV photon escaping from 1.1 µm material	Solid angle of APD (sr)	APD efficiency (100 µm Si at 6.4 keV)	Fraction of time window used in APD†
0.89	0.3	0.95	0.21	0.93	0.64

† Time window 23–120 ns.

## Footnotes

1 Supplementary data for this paper are available from the IUCr electronic archives (Reference: HF5209). Services for accessing these data are described at the back of the journal.
