# Supplementary material for: Exploration of synchrotron Mössbauer microscopy with micrometer resolution: forward and a new backscattering modality on natural samples
Source: J Synchrotron Radiat. 2012 Aug 8;19(Pt 5):814–20. doi: 10.1107/S0909049512032414 (PMC3423314; doi:10.1107/S0909049512032414)
Supplement: Supplementary file 1 [file s-19-00814-sup1.pdf]

1) Source characteristics (APS Science 2010)
Electron beam energy: 7GeV
Current: 100 mA
Type: undulator
Period: 2.7 cm
Length: 2 × 2.4 m
Tuning range: 6.7 -16.0 keV (1st harmonic)
Gap: 19.0 mm (for 14.4 keV)
Photon source properties (source size /source divergence):
Σ_x: 275 μm / $\Sigma_{x'}$: 12.3 μrad
Σ_y: 11.2 μm / $\Sigma_{y'}$: 4.7 μrad
On-axis spectral brilliance at 12 keV: 5.7×10^{19} ph/s/mrad^2/mm^2/0.1%bw

2) Components position from source

	Position from source (m)	Flux (ph/s)	Bandwidth (eV)
HHLM	29.5	2×10^{13} in 1 eV	1
HRM	34.2	5×10^{9} in 1 meV	1×10^{-3}
KB focusing mirror	35.2 (V mirror),<br>35.7 (H mirror)	1.5×10^{9} in 1 meV	1×10^{-3}
Pinhole	36.2	6×10^{7} in 1 meV	1×10^{-3}
Sample	36.2	--	1×10^{-3}

3) HRM used for ^{57}Fe at 14.4 keV, in-line (+ – – +) scattering geometry (Toellner, unpublished)

Channel-cut crystals	Si(4 0 0)		Si(10 6 4)	
Bragg angle (deg)	18.47		77.45	
2d (Å), (d: lattice distance)	2.71551		0.88103	
Asymmetric factor	0.264	0.076	1.354	36.299
Flux (ph/s)	~ 3×10^{12} in bandwidth of 0.3 eV, after 1st pair of Si (4 0 0)		5×10^{9} in 1 meV bandwidth, after 2nd pair of Si (10 6 4)	

4) KB focusing mirror at 14.4 keV

	Vertical focusing mirror	Horizontal focusing mirror (16 segments)
Mirror size in length (mm)	200	600
Mirror materials	polished Zerodur glass substrates	fused silica
Coating materials	Rh coating (1200 Å),<br>Cr binding layer (50 Å)	Pd coating (975 Å),<br>Cr binding layer (50 Å)
Reflectivity	85%	85%
Bending mechanic	mechanical bender	piezoelectric bimorph
Spatial acceptance (mm)	0.44	1.32
Incidence angle (mrad)	2.2	2.2
Distance to focal spot (mm)	990	500
Focused beamsize (μm)	18	20
Divergence of focal beam (mrad)	0.4	2.4

5) Others
HHLM at 14.4 keV: diamond (1 1 1) with lattice distance 2.05938 Å, Bragg angle 12.05 deg.
Pinhole: Stainless-steel arm, Pt with 5 μm aperture.

References

APS Science 2010, ANL-10/35, pp146.
Toellner, T. S. Argonne National Laboratory. *unpublished*.
